# Nicotine promotes the development of non-small cell lung cancer through activating LINC00460 and PI3K/Akt signaling

**DOI:** 10.1042/BSR20182443

**Published:** 2019-06-07

**Authors:** Hongying Zhao, Yu Wang, Xiubao Ren

**Affiliations:** 1 Oncology Department, Tianjin Medical University Cancer Institute and Hospital, Tianjin, P.R. China; 2 Department of Gerneral Surgery, Xuzhou Cancer Hospital, Xuzhou, 221000 Jiangsu, P.R. China

**Keywords:** apoptosis, cell migration, nicotine, non-small cell lung cancer, LINC00460, proliferation

## Abstract

**Objective**: Nicotine, the main ingredient in tobacco, is identified to facilitate tumorigenesis and accelerate metastasis in tumor. Studies in recent years have reported that long intergenic non-protein coding RNA 460 (LINC00460) is strongly associated with lung cancer poor prognosis and nicotine dependence. Nonetheless, it is unclear whether nicotine promotes the development of lung cancer through activation of LINC00460. **Methods:** We determined that LINC00460 expression in lung cancer tissues and the prognosis in patients with non-small cell lung carcinoma (NSCLC) using Gene Expression Profiling Interactive Analysis (GEPIA) website and The Cancer Genome Atlas (TCGA) database. Through *in vitro* experiments, we studied the effects of nicotine on LINC00460 in NSCLC cells lines using Cell Counting Kit-8 (CCK-8), transwell test, flow cytometry, quantitative reverse-transcription polymerase chain reaction (qRT-PCR) and Western blot assays. **Results**: We identified the significant up-regulated expression level of LINC00460 in NSCLC tissues and cell lines, especially, the negative correlation of LINC00460 expression level with overall survival (OS). In *in vitro* experiments, LINC00460 was overexpressed in NSCLC cell lines under nicotine stimulation. Nicotine could relieve the effect of LINC00460 knockdown on NSCLC cell proliferation, migration and apoptosis. The same influence was observed on PI3K/Akt signaling pathway. **Conclusions**: In summary, this is the first time to examine the potential roles of LINC00460 in lung cancer cell proliferation, migration and apoptosis induced by nicotine. This may help to develop novel therapeutic strategies for the prevention and treatment of metastatic tumors from cigarette smoke-caused lung cancer by blocking the nicotine-activated LINC00460 pathway.

## Introduction

It is well known that mortality caused by lung cancer ranks first in cancer-related deaths worldwide [1]. Lung cancer mainly includes two types, namely non-small cell lung cancer (NSCLC) and small cell lung cancer, the former of which accounts for approximately 85% in lung cancer [2,3]. Moreover, cigarette smoking comprises almost 90% of all deaths from lung cancer [4,5]. In addition to its documented risks for lung carcinogenesis, cigarette smoking has also been implicated in cancer development. Recently, Izzotti et al. [6] investigated the expression of 484 microRNAs (miRNAs) in the rat lungs exposed to environmental cigarette smoke for 4 weeks, found that cigarette smoke down-regulates 126 miRNAs at least two-fold and 24 miRNAs more than three-fold. Their dysregulation is associated with the initiation and development of lung cancer, which suggested that tobacco smoke might exert their carcinogenic effects by deregulation of miRNAs [7,8]. Nicotine is a major alkaloid in tobacco plants. Its biosynthesis and regulation in tobacco (*Nicotiana tabacum*) have been well studied.

Long non-coding RNAs (lncRNAs) can participate in the occurrence and development of tumor functioning as oncogenes or tumor suppressor [9–11]. Long intergenic non-protein coding RNA 460 (LINC00460) is one of lncRNAs and shows a high expression level in squamous-cell carcinoma and esophageal cancer cells [12]. Besides, it is associated with poor prognosis in patients with squamous-cell carcinoma of the head and neck, as well as tumor node metastasis (TNM) staging and lymph node metastasis [13]. Similarly, the LINC00460 has a higher expression level in NSCLC tissues and cells and interference of LINC00460 can affect cell invasion and migration of NSCLC via regulating epithelial–mesenchymal transition (EMT) [14–16]. Many chemical and physical agents have been reported to induce or treat a disease via dysregulating miRNAs or restoration of aberrant miRNAs [17], respectively. However, there are few reports on the involvement of non-coding RNAs in alkaloid biosynthesis.

In the present study, we determined that LINC00460 was overexpressed in lung cancer tissues, which led to a poor prognosis in patients with NSCLC. Through *in vitro* experiments, we first examined the effects of nicotine on LINC00460 in NSCLC cells lines and investigated the underlying mechanism.

## Materials and methods

### Database analysis

The RNA sequencing expression data of LINC00460 was downloaded from Gene Expression Profiling Interactive Analysis (GEPIA, http://gepia.cancer-pku.cn/) [18], which analyzed 483 NSCLC tumors and 347 normal samples from The Cancer Genome Atlas (TCGA, https://cancergenome.nih.gov/) database. Besides, under the influence of different LINC00460 expression level, the overall survival (OS) of patients with NSCLC was also analyzed.

### Cell culture

The NSCLC cell lines A549 and H1299 were all obtained from Chinese Academy of Sciences Shanghai Branch Cell Bank (Shanghai, China). A549 cells were cultured in F12K culture medium (Sigma–Aldrich, St. Louis, MO, U.S.A.) supplemented with 2.5 g/l NaHCO_3_ and 10% (v/v) fetal bovine serum (FBS; Sigma–Aldrich), while H1299 cells were seeded in Roswell Park Memorial Institute (RPMI) 1640 culture medium (Sigma–Aldrich) supplemented with 1.5 g/l NaHCO_3_, 2.5 g/l glucose, 0.11 g/l sodium pyruvate and 10 % (v/v) FBS at 37°C in 5% CO_2_ atmosphere. Until the cells grew into logarithmic phase, they were washed three times by PBS, digested with trypsin and then repeatedly beaten to single cell suspension. Subsequently, cells were seeded into a six-well plate in preparation for the following experiments.

Nicotine was purchased from Sigma–Aldrich (product number N3876) and added to cells after dilution with a concentration of 0, 1, 10, 100 and 200 μg/ml. After 48 h, LINC00460 expression level in A549 and H1299 cells was detected using quantitative reverse-transcription polymerase chain reaction (qRT-PCR). Time-dependent analysis was also measured as above instructions using 100 μg/ml.

### RNA interference

When cells grew to logarithmic phase, the medium was replaced with fresh medium without serum and antibiotics 2 h prior to transfection. The short hairpin RNA (sh-RNA) (5′-UCACCUUGACUACUGCUAUTT-3′) was chemically synthesized from Genepharma Co. Ltd. (Shanghai, China) and added with Lipofectamine 2000 (Invitrogen, Carlsbad, CA, U.S.A.) into cells according to the manufacturer’s instructions. After 24 h, the expression of pSUPER-LINC00460 (LINC00460-KD) mRNA in transfected cells could be observed using qRT-PCR.

### QRT-PCR assay

Total RNA of cells was extracted using 1.0 ml TRIzol (Invitrogen) after transfection of 48 h. Synthesis of cDNA was conducted with reverse transcriptase M-MLV (Takara Bio Inc., Shiga, Japan). qRT-PCR was used to detect the expression level of LINC00460 using SYBR Premix ExTaq™ kit (Applied Biosystems, Foster City, CA, U.S.A.) following the manufacturer’s instructions and the parameters were as follows: hot start at 95°C for 5 min; followed by 40 cycles of 95°C for 30 s, 60°C for 45 s and 72°C for 30 min. The primer sequence was as follows: LINC00460, F: 5′-ATGCACACTTCTCGGCTAAG-3′, R: 5′-GGTCGTAACCTTCGTTCTCATC-3′; β-actin (NC, negative control), F: 5′-CCCGAGCCGTGTTTCCT-3′, R: 5′-GTCCCAGTTGGTGACGATGC-3′. The relative quantitation of the value was determined using the 2^−ΔΔ*C*^_t_ calculation method and each sample was assayed in triplicates.

### Cell proliferation assay

According to the manufacturer’s protocols, cell viability was detected using Cell Counting Kit-8 (CCK-8; Beijing Solarbio Science & Technology Co., Ltd., Beijing, China) assay after transfection for 24 h. A total of 100 μl cell suspension (1000 cells/well) was placed in 96-well plates and after 10 μl of CCK-8 reagent was added to each well, their viabilities were detected at 0, 24, 48, 72 h. Optical density (OD) value was measured using a microplate reader (Bio-Rad, Hercules, CA, U.S.A.) at a wavelength of 450 nm. The experiments were performed in triplications.

### Cell migration assay

Transwell assay was used to detect cell migration utilizing A549 and H1299 cell lines. Based on the manufacturer’s instructions, 24-well transwell chamber with Matrigel matrix (BD Biosciences, Franklin Lakes, NJ, U.S.A.) was performed for cell invasion. A density of 5 × 10^3^ cells after transfection for 24 h was added into the upper chamber and 500 μl complete medium was added into the bottom chamber. After overnight, cells were washed by PBS, fixed with 4% paraformaldehyde for 30 min and stained with 0.1% Crystal Violet for 20 min. The number of migrated cells was counted under a microscope in five random fields per well at 200× magnification.

### Cell apoptosis assay

Flow cytometry was used to detect apoptotic cells. After transfection for 24 h, cells were trypsinized without ethylenediaminetetraacetic acid (EDTA) and collected in a centrifuge tube, then the cells were centrifuged at 1000 rpm for 5 min. Afterward, cells were resuspended in PBS pre-cooled at 4°C, centrifuged again and carefully aspirated the supernatant. After that we added 1× binding buffer to resuspend the cells and regulated the cell density of 1–5 × 10^6^/ml. One hundred microliters of the cells were gently mixed with 5 μl of Annexin V-FITC/PI (Beijing 4A Biotech Co., Ltd, Beijing, China) and incubated in the dark for 5 min at room temperature. Ten microliters of PI dye and 400 μl of PBS were added to the samples, and the results were analyzed in a flow cytometer using FlowJo. The experiment was repeated three times independently.

### Western blot analysis

After transfection for 48 h, total proteins were extracted by a Radio Immunoprecipitation Assay (RIPA) lysis buffer (Beyotime Inc., Shanghai, China) and quantitated by a BCA Protein Assay Kit (Beyotime Inc.) for the concentrations. Cell lysates (20 µg protein/lane) were separated by 8–12% sodium dodecyl sulfate/polyacrylamide gel electrophoresis (SDS/PAGE) and then transferred on to polyvinylidene fluoride (PVDF) membrane. After blocking with 5% non-fat dry milk for 1 h, the membranes were incubated with primary antibodies (1:1000; Cell Signaling Technology Inc., Danvers, MA, U.S.A.) overnight at 4°C. The primary antibodies were used against protein kinase B (AKT), p-AKT, phosphorylated-serine-threonine protein kinase (p-P70S6K), Cyclin D1, B-cell lymphoma 2 (Bcl-2), Bcl-2-associated X protein (Bax), Cleaved Caspase-3 and Glyceraldehyde 3-phosphate dehydrogenase (GAPDH). Followed by washing for 3–5 min, the membranes were incubated by anti-mouse/rabbit horseradish peroxidase–conjugated secondary antibodies (1:3000; Cell Signaling Technology Inc.) at room temperature and detected with electro-chemi-luminescence (ECL) imaging analysis system (Thermo Fisher Scientific Inc, Waltham, MA, U.S.A.). The bands were scanned with Quantity One software (Bio-Rad).

### Statistical analysis

All statistical analyses were performed by SPSS 22.0 (SPSS Inc., Chicago, IL, U.S.A.) software. The comparison of two groups used Student’s *t* test and more than two groups used Analysis of variance (ANOVA). The data were presented as mean ± standard deviation (SD) and a value of *P*<0.05 was considered statistically significant.

## Results

### LINC00460 was overexpressed in NSCLC tissues and led to worse OS

**Figure 1:**
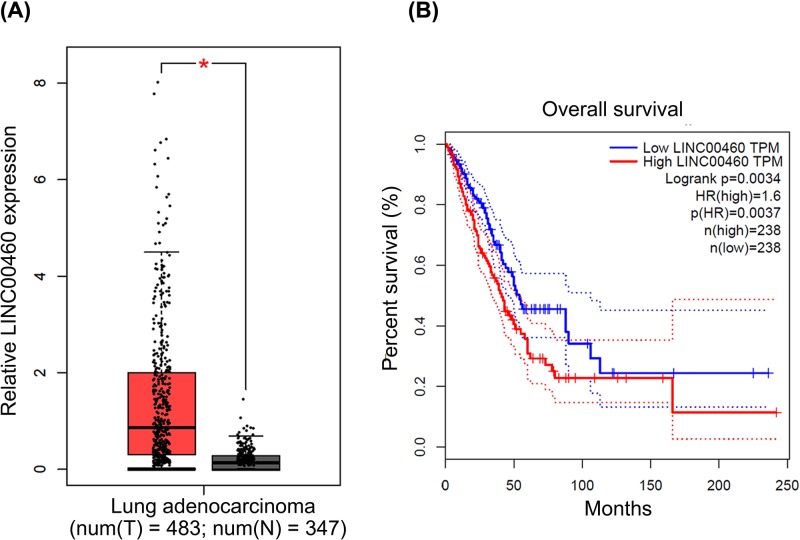
Expression of LINC00460 in NSCLC tissues based on GEPIA data (**A**) Expression levels of LINC00460 in NSCLC tissues (*n*=483) and normal tissue (*n*=347) from the TCGA database were analyzed. *P<0.05. (**B**) OS after 20 years of patients with low or high expression levels of LINC00460 was analyzed by Kaplan–Meier method and log rank test. *P*=0.0037.

First, we learned from the data of GEPIA that LINC00460 was overexpressed in lung adenocarcinoma tissues compared with the normal tissues (Figure 1A, *P<0.05). Besides, percent survival of NSCLC patients with LINC00460 high expression was significantly lower than that of patients with LINC00460 low expression (Figure 1B, *P*=0.0037). Therefore, it could be remarkably found that overexpression of LINC00460 exerted poor prognosis of the NSCLC patients, implying LINC00460 may be a potential prognostic marker for patients with advanced NSCLC.

### LINC00460 was up-regulated in NSCLC cell lines under the nicotine stimulation

**Figure 2:**
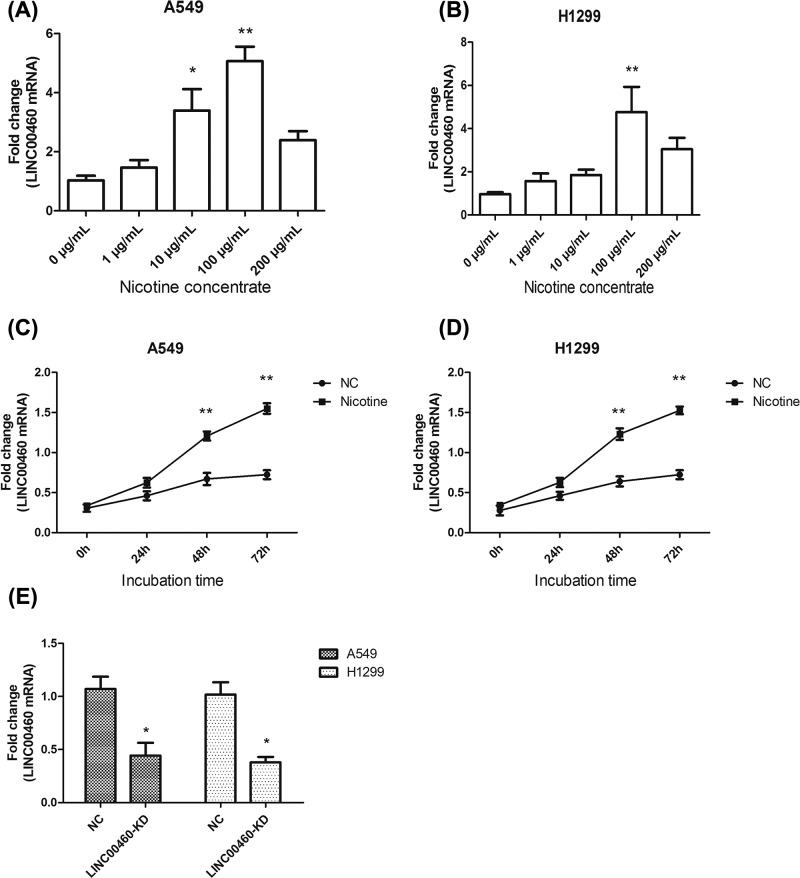
Expression of LINC00460 in NSCLC cell lines under nicotine induced (**A,B**) Expression levels of LINC00460 in NSCLC cell lines (A549 and H1299) under nicotine stimulation were measured by qRT-PCR. *P<0.05, **P<0.01 *vs.* 0 μg/ml group. (**C,D**) The time-dependent analysis of LINC00460 expression levels in NSCLC cell lines (A549 and H1299). **P<0.01 *vs.* NC group. (**E**) The expression levels of LINC00460 knockdown mRNA were assessed using qRT-PCR, *P<0.05 *vs.* NC group. LINC00460-KD, small interfering LINC00460 RNA.

In view of the previous analysis, we then explored the expression of LINC00460 in NSCLC cell lines, especially under the nicotine stimulation. We detected its expression in A549 and H1299 cell lines under different concentrations (0, 1, 10, 100, 200 μg/ml) of nicotine using qRT-PCR. As shown in the Figure 2A,B, LINC00460 expression was increased to various degrees, notably under the stimulation of 100 μg/ml nicotine in both the cell lines (*P<0.05, **P<0.01). Besides, we also assessed the time-dependent analysis using 100 μg/ml nicotine to observe the expression level of LINC00460. The expression level of LINC00460 is significantly higher than that of the control group (Figure 2C,D, **P<0.01). Hence, we chose this concentration of 100 μg/ml to perform the following experiments.

Subsequently, in order to verify the direct effect of nicotine on LINC00460, sh-RNA was used to silence LINC00460 and the mRNA expression level was detected by qRT-PCR after 72 h transfection. In Figure 2E, the LINC00460 expression was both decreased markedly in A549 and H1299 cell lines (*P<0.05). Thus we could confirm that LINC00460 was induced by nicotine.

### The effect of LINC00460 on cell proliferation was mediated by nicotine

**Figure 3:**
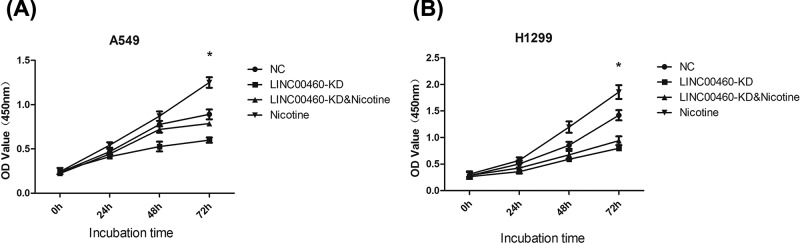
Effect of LINC00460 knockdown on NSCLC cells proliferation (**A**) OD values in A549 cell were detected by CCK-8 assay in three groups for 0, 24, 48, 72 h. *P<0.05 *vs.* NC group. (**B**) OD values in H1299 cell were detected by CCK-8 assay in three groups for 0, 24, 48, 72 h. *P<0.05 *vs.* NC group.

The effect of LINC00460 knockdown on proliferation of A549 and H1299 cells was assessed using CCK-8 assay. As shown in Figure 3A, the OD values of A549 cells in LINC00460-KD group decreased at 72 h compared with those in NC or nicotine group (*P<0.05), suggesting knockdown of LINC00460 could inhibit the proliferation of NSCLC cells. However, the inhibition was escaped from the nicotine addition (*P<0.05). Similar results were observed in H1299 cells (Figure 3B, *P<0.05). In general, these results indicated that LINC00460 could promote cell proliferation which could be encouraged by nicotine in NSCLC cells.

### Nicotine restored the inhibitory effect of LINC00460 knockdown on NSCLC cell migration

**Figure 4:**
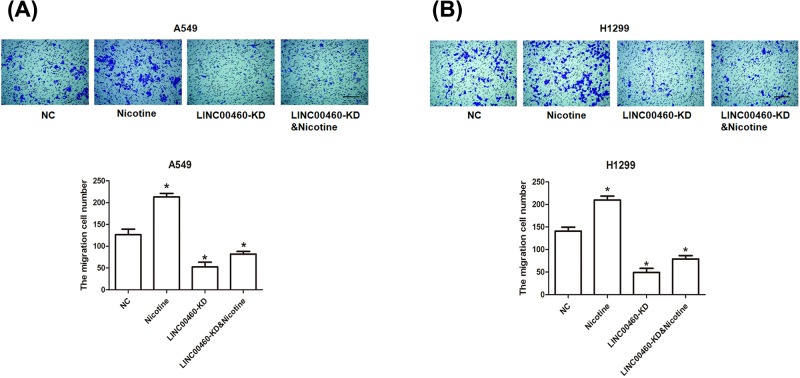
Effect of LINC00460 knockdown on NSCLC cell migration (**A**) Microscopic images of A549 cells passing though the microwells of the transwell chamber were detected by transwell assay. The number of migrated cells was counted. *P<0.05 *vs.* NC group, bar = 200 μm. (**B**) Microscopic images of H1299 cells passing though the microwells of the transwell chamber were detected by transwell assay. The number of migrated cells was counted. *P<0.05 *vs.* NC group, bar = 200 μm.

To further confirm the role of LINC00460 on the migration of NSCLC cells, transwell assay was used to determine the cell migratory ability induced by the treatment of nicotine after silencing LINC00460 in A549 and H1299 cells. In Figure 4A, A549 cells significantly lost their migratory capability after LINC00460 knockdown (*P<0.05), while under the treatment of nicotine, cells once again acquired the migrated ability to some degree (*P<0.05). The nicotine-alone group was applied to show its promoted effect on cell migration. Likewise, the similar tendency in migration was revealed in H1299 cells because of nicotine interference (Figure 4B, *P<0.05). Collectively, LINC00460 knockdown plays an inhibitory role in NSCLC cell migration, which can be attenuated by nicotine.

### Nicotine was involved in cell apoptosis mediated by LINC00460

**Figure 5:**
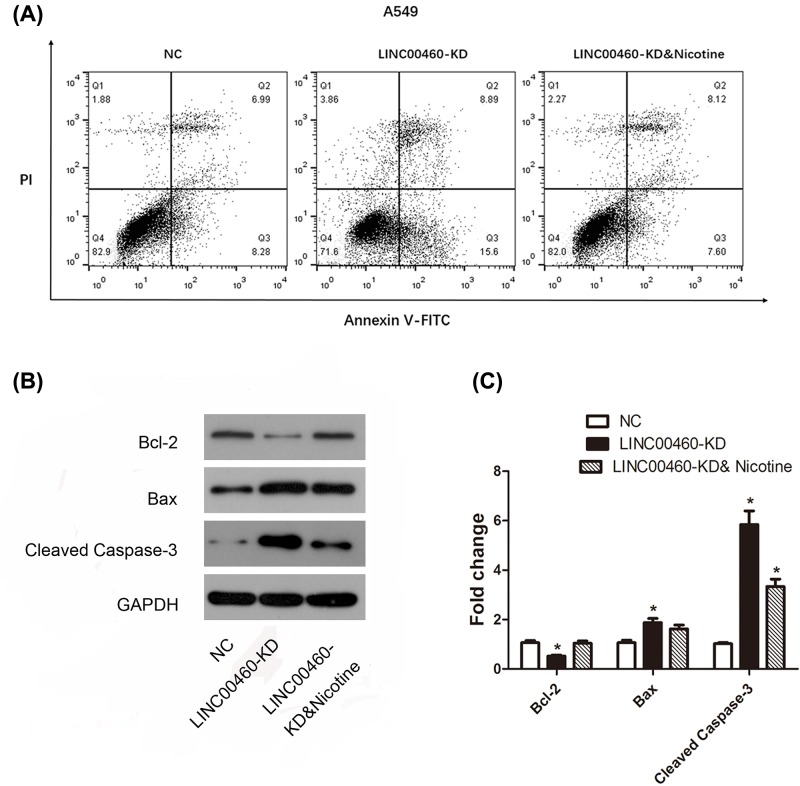
Effect of LINC00460 knockdown on apoptosis in NSCLC A549 cells (**A**) Flow cytometric plots show specific cell populations in A549 cells. Q2 were late apoptotic cells, Q3 were live cells, and Q4 were early apoptotic cells. (**B**) Expression of activated Bcl-2, Bax and Cleaved Caspase-3 were determined by Western blot and shown by bar graphs in (**C**). *P<0.05 *vs.* NC group. Data were expressed as mean ± SD.

**Figure 6:**
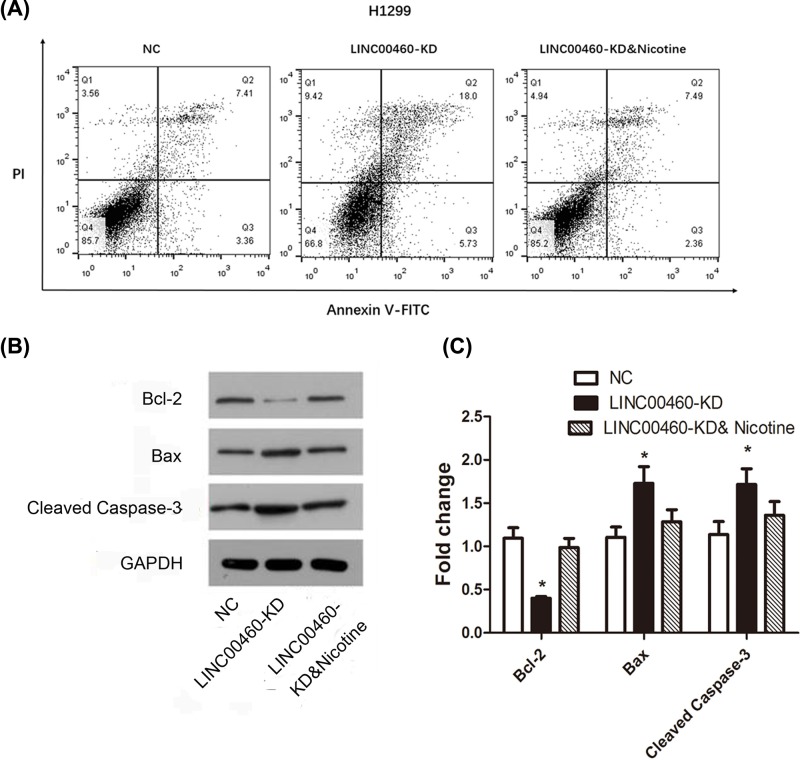
Effect of LINC00460 knockdown on apoptosis in NSCLC H1299 cells (**A**) Flow cytometric plots show specific cell populations in H1299 cells. Q2 were late apoptotic cells, Q3 were live cells, and Q4 were early apoptotic cells. (**B**) Expression of activated Bcl-2, Bax and Caspase3 were determined by Western blot and showed by bar graphs in (**C**). *P<0.05 *vs.* NC group.

Flow cytometry after Annexin V-FITC/PI staining and Western blot assay were used to explore whether LINC00460 knockdown was associated with cell apoptosis. Results in Figure 5 revealed that compared with NC group, the expression level of anti-apoptotic protein Bcl-2 was decreased after LINC00460 knockdown, conversely, pro-apoptotic protein Bax and Cleaved Caspase-3 expression were increased in A549 cells (*P<0.05). As expected, nicotine rescued the suppressive effect of LINC00460 knockdown on apoptosis. The same results were established in H1299 cells (Figure 6, all *P<0.05). As a consequence, silencing LINC00460 impaired cellular apoptotic prosperity in NSCLC cells but nicotine significantly relieved this impact.

### Nicotine could relieve suppressive effect of LINC00460 knockdown on PI3K/Akt signaling pathway

**Figure 7:**
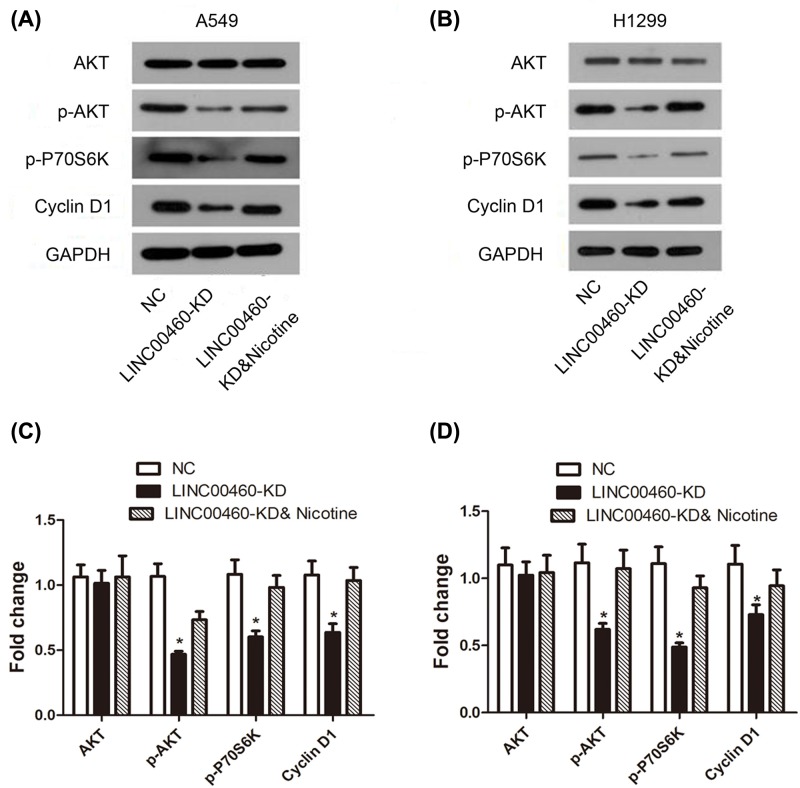
Effect of LINC00460 knockdown on activation of PI3K pathway in NSCLC cell lines (**A,B**) Represented bands of PI3K/Akt signaling associated proteins were assessed by Western blot in A549 and H1299 cells. (**C,D**) Relative protein expression levels of proteins were showed by column diagram. *P<0.05 *vs.* NC group.

In order to explore the underlying mechanism of LINC00460 in NSCLC cell, we determined the protein expression level of PI3K/Akt signaling-related markers by Western blot assay (Figure 7A,B). The results showed that expression levels of p-AKT, p-P70S6K and Cyclin D1 in LINC00460-KD group were significantly decreased compared with NC group (Figure 7C,D, *P<0.05), while the reduced expression was restored under nicotine stimulation. The data showed that the PI3K/Akt signaling pathway was significantly inhibited by LINC00460 knockdown, and the suppressive effect was eliminated by nicotine.

## Discussion

In this present investigation, we identified that the effect of LINC00460 knockdown on NSCLC cells’ activities can be restored by administration of nicotine and regulating the PI3K/Akt signaling pathway. All the findings established that the significance of LINC00460 activation induced by nicotine in NSCLC cell behaviors, enriching awareness of tumor treatments relying on lncRNAs.

Cigarette smoking has been found to enhance dissemination of tumor cells in the body of patients with various types of cancer [4,5,15]. Nicotine, one of the major components of cigarette smoking, has a role on cell proliferation of colorectal cancer, as well as of cell lines derived from several different cancer types [19]. It can suppress cell apoptosis induced by extracellular stress stimuli both in normal cells, such as vascular endothelial and smooth muscle cells [5], and in several human cancer cell lines derived from various organs [4,20]. Although there are a few studies on the mechanism of nicotine carcinogenesis, the effects of nicotine on lung cancer is still needed to fulfill in detail. In our *in vitro* experiments, LINC00460 was up-regulated in NSCLC cell lines owing to the nicotine stimulation. Nicotine can relieve the inhibition of LINC00460 knockdown on NSCLC cell proliferation and migration. Moreover, the effect of LINC00460 knockdown on NSCLC cell apoptosis was also related to nicotine, along with the alteration of Bcl-2, Bax and Cleaved Caspase-3. Furthermore, nicotine can make the suppressive effect of LINC00460 knockdown on PI3K/Akt signaling pathway worse. Thus, we boldly speculated that nicotine’s regulation of LINC00460 might play an important role in the development of NSCLC.

As reported in various papers, nicotine activates multiple survival mechanisms, including PI3K/Akt and PKC/ERK1/2 pathways [9]. We also found that nicotine can make the suppressive effect of LINC00460 knockdown on PI3K signaling pathway worse. The PI3K/Akt signaling pathway is crucial for normal cell growth, and its deregulation influences various cellular responses that are associated with cancer phenotypes [21–26]. It runs as following steps: at first, PI3K activation phosphorylates AKT and active AKT can lead to a number of downstream effects including the activation of mTOR, ultimately mediate p-P70S6K activity, which in turn directly impacts cell growth and survival [27–29]. The main influence of the activation of Akt is the increase in survival in cell that normally undergoes death by apoptosis [19] and is involved in many other progressions, such as cell proliferation, angiogenesis, invasiveness and migration, modulating the initiation and progression of cancer [30,31]. Therefore, in accordance with above-mentioned reports, we hypothesized that LINC00460 might act as one upstream of PI3K/Akt pathway to control NSCLC progression. Furthermore, the consistent findings have been explained by Ma et al. [32], indicating that LINC00460 could promote tumor growth and gefitinib resistance via EMT [33,34].

In conclusion, to the best of our knowledge, this is the first study to reveal the potential roles of LINC00460 in lung cancer cell growth, migration and apoptosis induced by nicotine. This may help to develop novel therapeutic strategies for the prevention and treatment of lung cancer caused by cigarette smoke through inhibiting the nicotine-activated LINC00460 pathways.

## Abbreviations

AKT: protein kinase B
Bax: Bcl-2-associated X protein
Bcl-2: B-cell lymphoma 2
CCK-8: cell counting kit-8
EMT: epithelial–mesenchymal transition
FBS: fetal bovine serum
F12K culture medium: Kaighn’s Modification of Ham’s F-12 Medium
GEPIA: Gene Expression Profiling Interactive Analysis
LINC00460: long intergenic non-protein coding RNA 460
lncRNA: long non-coding RNA
miRNA: microRNA
NC: negative control
NSCLC: non-small cell lung carcinoma
OD: optical density
OS: overall survival
PI: Ppidium Iodide
PKC/ERK1/2: protein kinases C (PKC)/extracellular-regulated protein kinases 1/2
p-P70S6K: phosphorylated-serine-threonine protein kinase
qRT-PCR: quantitative reverse-transcription polymerase chain reaction
sh-RNA: short hairpin RNA
TCGA: The Cancer Genome Atlas
TNM: tumor node metastasis

## References

[1] Bray F., Ferlay J., Soerjomataram I., Siegel R.L., Torre L.A. and Jemal A. (2018) Global cancer statistics 2018: Globocan estimates of incidence and mortality worldwide for 36 cancers in 185 countries. CA Cancer J. Clin. 68, 394–424 10.3322/caac.21492 30207593

[2] Cui S., Zhang W., Xiong L., Pan F., Niu Y., Chu T. (2017) Use of capture-based next-generation sequencing to detect Alk fusion in plasma cell-free DNA of patients with non-small-cell lung cancer. Oncotarget 8, 2771–2780 27926526 10.18632/oncotarget.13741 PMC5356840

[3] Zhao Q., Gu X., Zhang C., Lu Q., Chen H. and Xu L. (2015) Blocking M2 muscarinic receptor signaling inhibits tumor growth and reverses epithelial-mesenchymal transition (Emt) in non-small cell lung cancer (Nsclc). Cancer Biol. Ther. 16, 634–643 10.1080/15384047.2015.1029835 25778781 PMC4622973

[4] Du X., Qi F., Lu S., Li Y. and Han W. (2018) Nicotine upregulates Fgfr3 and Rb1 expression and promotes non-small cell lung cancer cell proliferation and epithelial-to-mesenchymal transition via downregulation of mir-99b and mir-192. Biomed. Pharmacother. 101, 656–662 10.1016/j.biopha.2018.02.113 29518612

[5] Greillier L., Cortot A.B., Viguier J., Brignoli-Guibaudet L., Lhomel C., Eisinger F. (2018) Perception of lung cancer risk: impact of smoking status and nicotine dependence. Curr. Oncol. Rep. 20, 18 10.1007/s11912-017-0650-1 29508085

[6] Izzotti A., Calin G.A., Arrigo P., Steele V.E., Croce C.M. and De Flora S. (2009) Downregulation of microRNA expression in the lungs of rats exposed to cigarette smoke. FASEB J. 23, 806–812 10.1096/fj.08-121384 18952709 PMC2653990

[7] Sun H. and Ma X. (2015) Alpha5-Nachr modulates nicotine-induced cell migration and invasion in A549 lung cancer cells. Exp. Toxicol. Pathol. 67, 477–482 10.1016/j.etp.2015.07.001 26205096

[8] Marsit C.J., Eddy K. and Kelsey K.T. (2006) MicroRNA responses to cellular stress. Cancer Res. 66, 10843–10848 10.1158/0008-5472.CAN-06-1894 17108120

[9] Kong Y.G., Cui M., Chen S.M., Xu Y., Xu Y. and Tao Z.Z. (2018) LncRNA-Linc00460 facilitates nasopharyngeal carcinoma tumorigenesis through sponging miR-149-5p to up-regulate IL6. Gene 639, 77–84 10.1016/j.gene.2017.10.006 28987345

[10] Li L., Wang M., Mei Z., Cao W., Yang Y., Wang Y. (2017) LncRNAs Hif1a-As2 facilitates the up-regulation of Hif-1alpha by sponging to miR-153-3p, whereby promoting angiogenesis in Huvecs in hypoxia. Biomed. Pharmacother. 96, 165–172 10.1016/j.biopha.2017.09.113 28985553

[11] Webb A., Papp A.C., Curtis A., Newman L.C., Pietrzak M., Seweryn M. (2015) RNA sequencing of transcriptomes in human brain regions: protein-coding and non-coding RNAs, isoforms and alleles. BMC Genomics 16, 990 10.1186/s12864-015-2207-8 26597164 PMC4657279

[12] Huang G.W., Xue Y.J., Wu Z.Y., Xu X.E., Wu J.Y., Cao H.H. (2018) A three-LncRNA signature predicts overall survival and disease-free survival in patients with esophageal squamous cell carcinoma. BMC Cancer 18, 147 10.1186/s12885-018-4058-6 29409459 PMC5801805

[13] Liang Y., Wu Y., Chen X., Zhang S., Wang K., Guan X. (2017) A novel long noncoding RNA Linc00460 up-regulated by Cbp/P300 Promotes carcinogenesis in esophageal squamous cell carcinoma. Biosci. Rep. 37, BSR20171019, 10.1042/BSR20171019 28939763 PMC5964888

[14] Yue Q.-Y. and Zhang Y. (2018) Effects of Linc00460 on cell migration and invasion through regulating epithelial-mesenchymal transition (Emt) in non-small cell lung cancer. Eur. Rev. Med. Pharmacol. Sci. 22, 1003–1010 29509248 10.26355/eurrev_201802_14382

[15] Li K., Sun D., Gou Q., Ke X., Gong Y., Zuo Y. (2018) Long non-coding RNA Linc00460 promotes epithelial-mesenchymal transition and cell migration in lung cancer cells. Cancer Lett. 420, 80–90 10.1016/j.canlet.2018.01.060 29409808

[16] Ye X. and Weinberg R.A. (2015) Epithelial-mesenchymal plasticity: a central regulator of cancer progression. Trends Cell Biol. 25, 675–686 10.1016/j.tcb.2015.07.012 26437589 PMC4628843

[17] Jiang Q., Wang Y., Hao Y., Juan L., Teng M., Zhang X. (2009) Mir2disease: a manually curated database for microrna deregulation in human disease. Nucleic Acids Res. 37, D98–D104 10.1093/nar/gkn714 18927107 PMC2686559

[18] Tang Z., Li C., Kang B., Gao G., Li C. and Zhang Z. (2017) Gepia: a web server for cancer and normal gene expression profiling and interactive analyses. Nucleic Acids Res. 45, W98–W102 10.1093/nar/gkx247 28407145 PMC5570223

[19] Cucina A., Dinicola S., Coluccia P., Proietti S., D’Anselmi F., Pasqualato A. (2012) Nicotine stimulates proliferation and inhibits apoptosis in colon cancer cell lines through activation of survival pathways. J. Surg. Res. 178, 233–241 10.1016/j.jss.2011.12.029 22520577

[20] Xie J. and Fan L. (2016) Nicotine biosynthesis is regulated by two more layers: small and long non-protein-coding RNAs. Plant Signal. Behav. 11, e1184811 10.1080/15592324.2016.1184811 27172239 PMC4973799

[21] Lee Y., Lee J.Y. and Kim M.H. (2014) Pi3k/Akt pathway regulates retinoic acid-induced hox gene expression in F9 cells. Dev. Growth Differ. 56, 518–525 10.1111/dgd.12152 25212816

[22] Holand K., Boller D., Hagel C., Dolski S., Treszl A., Pardo O.E. (2014) Targeting class Ia PI3K isoforms selectively impairs cell growth, survival, and migration in glioblastoma. PLoS ONE 9, e94132 10.1371/journal.pone.0094132 24718026 PMC3981776

[23] Thang N.D., Yajima I., Kumasaka M.Y., Iida M., Suzuki T. and Kato M. (2015) Deltex-3-like (Dtx3l) stimulates metastasis of melanoma through Fak/Pi3k/Akt but not Mek/Erk pathway. Oncotarget 6, 14290–14299 10.18632/oncotarget.3742 26033450 PMC4546467

[24] Arrighi N., Bodei S., Zani D., Michel M.C., Simeone C., Cosciani Cunico S. (2013) Different muscarinic receptor subtypes modulate proliferation of primary human detrusor smooth muscle cells via Akt/Pi3k and Map Kinases. Pharmacol. Res. 74, 1–6 10.1016/j.phrs.2013.04.007 23628881

[25] Fang J., Xia C., Cao Z., Zheng J.Z., Reed E. and Jiang B.H. (2005) Apigenin inhibits Vegf and Hif-1 expression via Pi3k/Akt/P70s6k1 and Hdm2/P53 pathways. FASEB J. 19, 342–353 10.1096/fj.04-2175com 15746177

[26] He D. and Zhang S. (2018) Unbs5162 inhibits the proliferation of esophageal cancer squamous cells via the Pi3k/Akt signaling pathway. Mol. Med. Rep. 17, 549–555 29115622 10.3892/mmr.2017.7893

[27] King D., Yeomanson D. and Bryant H.E. (2015) Pi3king the lock: targeting the Pi3k/Akt/Mtor pathway as a novel therapeutic strategy in neuroblastoma. J. Pediatr. Hematol. Oncol. 37, 245–251 10.1097/MPH.0000000000000329 25811750

[28] Peltier J., O’Neill A. and Schaffer D.V. (2007) Pi3k/Akt and Creb regulate adult neural hippocampal progenitor proliferation and differentiation. Dev. Neurobiol. 67, 1348–1361 10.1002/dneu.20506 17638387

[29] Rafalski V.A. and Brunet A. (2011) Energy metabolism in adult neural stem cell fate. Prog. Neurobiol. 93, 182–203 10.1016/j.pneurobio.2010.10.007 21056618

[30] Candeias E., Sebastiao I., Cardoso S., Carvalho C., Santos M.S., Oliveira C.R. (2018) Brain Glp-1/Igf-1 signaling and autophagy mediate Exendin-4 protection against apoptosis in type 2 diabetic rats. Mol. Neurobiol. 55, 4030–4050 28573460 10.1007/s12035-017-0622-3

[31] Jiang C., Ma S., Hu R., Wang X., Li M., Tian F. (2018) Effect of Cxcr4 on apoptosis in osteosarcoma cells via the Pi3k/Akt/Nf-kappabeta signaling pathway. Cell. Physiol. Biochem. 46, 2250–2260 10.1159/000489593 29734183

[32] Ma G., Zhu J., Liu F. and Yang Y. (2019) Long noncoding RNA Linc00460 promotes the Gefitinib resistance of non small cell lung cancer through epidermal growth factor receptor by sponging Mir-769-5p. DNA Cell Biol. 38, 176–183 10.1089/dna.2018.4462 30601026 PMC6383575

[33] Ye J.J., Cheng Y.L., Deng J.J., Tao W.P. and Wu L. (2019) Lncrna Linc00460 promotes tumor growth of human lung adenocarcinoma by targeting Mir-302c-5p/Foxa1 axis. Gene 685, 76–84 10.1016/j.gene.2018.10.058 30359741

[34] Yue Q.Y. and Zhang Y. (2018) Effects of Linc00460 on cell migration and invasion through regulating epithelial-mesenchymal transition (Emt) in non-small cell lung cancer. Eur. Rev. Med. Pharmacol. Sci. 22, 1003–1010 29509248 10.26355/eurrev_201802_14382

